# Conversations in couple relationships: a trustful foundation when making future parenthood “real”

**DOI:** 10.3389/fsoc.2024.1383028

**Published:** 2024-05-24

**Authors:** Marita Flisbäck

**Affiliations:** Department of Work Life and Social Welfare, Faculty of Caring Science, Work Life, and Social Welfare, University of Borås, Borås, Sweden

**Keywords:** existential imperative, parenthood, conversation, trust capital, nomos, existential motto, existential legitimacy, consensus

## Abstract

Sociologists often argue that communication in long-term couple relationships is the basis on which expectations, trust, and equality are created in contemporary society. However, what is the role of these everyday conversations in uncertain life situations such as expecting one’s first child? This article examines concerns reported by prospective Swedish parents in order to explain the role of communication to alleviate these. Concerns, related to the formation of new relationships with one’s partner, oneself, and one’s future child, are mitigated by referring to the couple’s “good” communication. In the present prenatal situation, the communication pattern (established in the past) seems to serve three functions in mitigating future concerns: (1) the communication generates a trust capital in the relationship, allowing the couple to venture into the uncertain future, (2) the communication makes social perceptions of family life “real” by constructing a common *nomos* that is internalized in the individual as an existential motto, and (3) the communication legitimizes family practices as democratic when referring to future plans as emerging from responsive and consensual dialogs. In the article it is emphasized that welfare policy needs to be based on an existential legitimacy, often developed in couple conversations, and particularly shaped in life situations characterized by change. However, the stability offered at the conversational micro level may simultaneously prevent macro level changes, a complexity that needs to be considered when developing a gender equality policy that is to resonate with people’s existential meaning making. With the aim of consensus, and the means of balancing conflicts, there is a risk that the conversation will consolidate the interests of the stronger party. In this way, the responsive conversations in long-term relationships may consolidate gender inequality and counteract the welfare policy goal of equalizing power relationships.

## Introduction

Becoming a parent raises a classical sociological question about the terms and means by which people adapt to, or manage to change, given social conditions (Berger, 1971). The change is most tangible for first-time parents, often with a stressful impact on the couple’s relationship [e.g., Demo and Cox (2000), Doss et al. (2009), and Fox (2009)]. Researchers have interpreted the transition as a life-changing event that can lead (heterosexual) couples to adopt a more traditional gender division of labor than they intend (Miller, 2011, 2017; Lévesque et al., 2020; Grunow and Evertsson, 2021). However, the outcome depends on factors as the cultural context, the couple’s education and material resources (Fox, 2009), and welfare state institutions (Goldscheider et al., 2015; Esping-Andersen, 2016). Also important are the responsive communication and the dynamic interaction formed between the parents, which seem to be shaped already in the prenatal phase [see, e.g., Ranta et al. (2023)].

In this article, I focus on the conversational interaction between parents in the prenatal phase. Starting from the premise that the long-term couple relationship is a social institution, which in contemporary society is increasingly based on conversations, trust, and expectations [see, e.g., Berger and Kellner (1964), Berger (1977), Giddens (1992), and Martin and Théry (2001)], I analyze the significance of conversations when future family relationships are established in the uncertain situation of expecting a first child. However, in order to understand the role of conversation when making future parenthood “real,” we first need to explore concerns prospective parents see on their future horizon. Therefore, the *first aim of this article* is to understand the main concerns that the interviewed, expectant parents depict in their imminent future in order to explain the role of communication to alleviate these fears. The empirical data consists of qualitative interviews with 25 expectant parents in Sweden. As we will see, in this prenatal phase, parents can be highly aware of the upcoming challenges but at the same time believe that a changed everyday life can be handled.

When analyzing the parallel concerns about future family practices, and the emphasis on the importance of communication as a tool to manage them, I make use of Berger’s phenomenological sociology. Berger is probably best known for the sociology of knowledge that he developed with Thomas Luckmann, in which communication in everyday life is understood as “the fabric of meanings without which no society could exist” (Berger and Luckmann, 1966, p. 15). In a classic text from Berger and Kellner (1964), the “marriage” is emphasized as the most important social institution to make sense of the social world and create a common meaningful order, i.e., a *nomos*. Within the framework of long-term partner relationships, individuals use dialogs and regular conversations to make the world comprehensible, “real,” and in this way: less uncertain [see also Berger (1977)]. In relation to the theoretical framework, *the second aim of this article* is to explore how this is done in a couple’s conversational practice when expecting a first child; in other words, to concretize and conceptually develop Berger’s theory by pointing out elements of the couple’s conversations on which the act of *the social construction of reality* is based.

In the section on theory and previous research that follows this introduction, I will develop Berger and Kellner’s perspective, and describe how the transition to parenthood has been interpreted and discussed in previous research. However, to more fully grasp how trustful dialogs are used in a particularly uncertain and life-changing situation, we need to take seriously the existential questions people ask themselves in similar circumstances (Berger, 1969, p. 93; Flisbäck and Bengtsson, 2024). Therefore, the concept *existential imperative,* developed by the social anthropologist Jackson (2005), also benefits the analysis. An existential imperative occurs in relation to crucial situations in which the openness of life becomes more evident than before as one’s routines do not continue as usual. In examining the trust-building role of communication in this regard, I make use of the concept of *trust capital* (Flisbäck, 2006, 2014), which denotes a crucial social resource for handling change, and existential, material, or social insecurity.

After theory and previous research, data and method will be presented, followed by the results. In the first empirical section, the prenatal phase as an existential imperative is highlighted. Then, I summarize three main concerns in the interviewees’ future horizon and describe how they mitigate these concerns by emphasizing the couple’s conversation as a source of stability in the transition from an old life to a new one. The context is Swedish society, which has traditionally been based on welfare policies aimed at equalizing both social and gender inequality (Esping-Andersen, 2002) to facilitate men’s and women’s adaptation to a new and more gender-equal family partnership (Goldscheider et al., 2015; Esping-Andersen, 2016). However, as Deutsch (2007) emphasizes, family and gender equality policies affect individuals, but the outcomes are always dependent on the concrete and communicative contexts in which interactions take place. In other words, to understand how parenthood is made intelligible in a phase characterized by uncertainty, and to explain the role of everyday conversations in this situation, also have significance at the macro level. I will return to this theme in the final discussion, which also contains the main conclusions.

## Theory and previous research

### Expecting a first child: an existential imperative with a changed approach to time

The challenge of parenthood is said to consist of “a new life and changed relationships” (Fox, 2009, p. 249). Research on the transition to parenthood is extensive, and has interpreted this phase as involving *embracing a new identity* as a mother or a father (Miller, 2005, 2017), *a rite de passage* (Lévesque et al., 2020), or *a biographical turning point* (Grunow and Evertsson, 2021). Using Jackson’s (2005) related concept, *existential imperative*, I analyze expecting a first child as a process in which fundamental questions about subjectivity, existential, and cultural meaning come to a head (Flisbäck, 2014). While a rite de passage concerns how people go through several transitional phases in life in which they try to find new social roles (van Gennep, 1960), Jackson, taking a phenomenological approach, points out that people in these situations are rarely shaped by roles prescribed in a cultural script. Existential imperatives are the result of the individual’s encounter with the world, and to some extent, the outcome of the event is always open [cf. Bengtsson and Flisbäck (2021)].

In existential imperatives, the finitude of life becomes more prominent than before and the experience of time more palpable. Emerging from the experience that one’s daily roles and routines soon will be changed, the meaning embedded in one’s current everyday life becomes visible and tangible, and awakens existential reflections on what one has done and wants to do in the future (Jackson, 2005). In other words, what I have done with the days in my past and what I intend to do in the future turn into central existential questions. This idea can be recognized from existential phenomenology, with for instance Heidegger (1996) stating that when something no longer functions as before, we might see the context of meaning that is otherwise hidden in our everyday life.

The imperative to reflect on existential issues creates potential for change but could also lead one to follow in the footsteps of previous generations (Jackson, 2005). As Berger (1971) once wrote: “Children are our hostages to history” (p. 4). Whether we like it or not, becoming a parent is a crucial social practice that binds us to tradition. When we worry, when we are short of time, or when our caring responsibilities become extensive, we often act in accordance with previous generations (see, e.g., Miller, 2011). However, the analysis in this article does not concern the outcome of the existential imperative of becoming a parent but rather the uncertainties that arise when expecting a first child and how the conversation is constructed as the couple’s main belief when handling these difficulties.

When analyzing the prenatal situation as an existential imperative, we will see how this life-changing situation produces a new approach to time whereby the past and the future are more intertwined than before. With the coming of parenthood, there are fears and hopes for a new future and sometimes, according to Altenburger et al. (2014), a retrospective examination of one’s own family of origin.

Neale et al. (2012, p. 4) state that “capturing imaginary futures /…/ is a powerful way to understand the changing aspirations of individuals.” Especially in longitudinal qualitative research, time is a key in which different *timescapes* have been analyzed in order to understand the construction of parenthood as a “temporal subjectivity” (Thomson, 2010; Miller, 2015, p. 295; Miller, 2017). Moreover, in relation to parenthood and gender inequalities, time has been frequently analyzed as a fundamental resource for exercising autonomy and power [see, e.g., Oakley (1979)]. Although the gendered nature of parenting has changed, mothers’ lack of autonomy persists as they still shoulder the “24/7 thinking responsibility of caring for children” (Miller, 2017, p. 146). In Sweden, despite gender equality policies and ideals, studies indicate that mothers still long for time of their own, beyond the constant commitments of caring for others and domestic work (Björnberg and Kollind, 2005). However, in the prenatal situation analyzed here, the changed approach to time is not primarily about time as an object of dreams, negotiation, scarcity, or power resource. Analyzed as an existential imperative, expecting a child gives rise to a “gap in time” in the flow between the past and the future [cf. Arendt (1978)]. With this tangible experience of being “betwixt and between” a new and an old life, the very meaning of time is changed [Jackson (2005) and Flisbäck (2014)]. In this situation there is a need for a belief that the past can be reconciled with the future, and this is where the couple’s conversation patterns may provide a trustful foundation.

### Managing uncertainty and developing trust capital

As we will see in the empirical sections, the existential imperative of expecting a child means facing something crucial and uncertain. As seen above, in similar situations questions of life meaning can emerge more prominently than before. Similar situations may pose a threat to the routines of everyday life, creating strong demands for individuals to jointly make sense of their upcoming new “reality” (Berger and Luckmann, 1966, p. 148–149; Berger, 1967). In their attempts to find order and trust in a shared reality, the language is fundamental (Berger, 1967). Here, Flisbäck (2006, 2014) has pointed out how *trust capital* can be a resource for managing uncertainty. Often shaped in the conversational practices of the everyday, and based on a voluntary relationship, trust capital provides support and tenderness [cf. Giddens (1992)].

*Trust* can be described as a social willingness to interact and communicate with others (Løgstrup, 2020). It is the link that binds us to others, promotes solidarity, and counteracts insecurity in social life (Durkheim, 1964). Building trust takes time, constituting a process in which expectations are both expressed, restrained, and balanced (Mauss, 2002; Théry, 2003). Therefore, the generation of trust capital requires closeness within the framework of a long-term relationship. The capital is thus exchanged and generated in kinship, a love relationship, friendship, or other close relationships. Its formation is based on the belief that recognition, support, care, and thoughtfulness are reciprocated in the long term, even if they are not immediately reimbursed [cf. Finch and Manson (1993), Mauss (2002), and Théry (2003)].

Like all forms of capital, the enabling effect of a trust capital becomes apparent to us when we seem to lose it, or when we are about to embark on something new and life-changing (Flisbäck, 2014). As a relational social resource for handling uncertainty, trust capital is a complement to Bourdieu’s (1989) cultural class analysis based on the possession of different forms of capital: economic, cultural, and social. Compared to social capital, trust capital provides emotional support that shapes our confidence in the future [cf. Nowotny (1981)]. Furthermore, trust capital has an impact on how other forms of capital are used. With self-confidence and faith in the future, we dare to embark on educational or professional routes whereby we enhance our cultural, social, and economic capital. In this way, trust capital may also counteract difficulties encountered in parenthood [cf. Wissö and Plantin (2015)], especially in the postnatal phase [cf. Fox (2009)]. In the prenatal phase, trust capital may support a belief that the future parenthood will be manageable. However, according to Løgstrup (2020), trust is always connected to the risk of one’s self-disclosure being exploited or treated with indifference, or of being left alone in one’s will to communicate. Recognizing the needs of the other and the vulnerability of trust is thus an important foundation in the formation of a trust capital, which also facilitates future experiences of coparenting, fair divisions of labor, and equal responsibilities [cf. Ranta et al. (2023)]. In this way the trust capital may provide a widened future horizon, grounded in a joint construction of reality.

### Conversation as a joint construction of reality

Today, long-term couple relationships are said to be increasingly based on ongoing communicative adjustments of balance and mutual expectation [see, e.g., Giddens (1992), Martin and Théry (2001)], an interaction in everyday life that is seen as a foundation for the development of democratic relationships, social equity, and gender equality [see, e.g., Habermas (1987), Giddens (1992), and Théry (2010)]. In other words, it is in the practice of communication with the people close to us that we not only understand and set the direction of our lives (Flisbäck, 2014) but also recognize the lives of others and society at large (Berger and Kellner, 1964; Berger, 1977).

Berger and Kellner’s (1964) theory on the construction of reality through a couple’s communication is useful in explaining how the parents’ “prenatal representations” come to be indicative of their future parenting [cf. Kuersten-Hogan (2017, p. 3)]. It also provides a deeper understanding of the ways of dealing with uncertainty and different concerns arisen in the prenatal situation, highlighted in this article.

According to Berger and Kellner, couples (in the context of long-term relationships) use everyday conversations to make the social world real and intelligible. As a central institution, the couple relationship creates an understanding of reality with shared values that mitigates existential and social ambiguity [see also Berger (1977)]. In conversation, individuals *externalize* their experiences, which are given a shared interpretation and are thus *objectified* and perceived as “real.” Furthermore, a common approach to the world is developed as a nomos that is then *internalized* in the individual, forming the basis of future orientations (Berger and Luckman, 1966).

From Berger’s (1977) perspective, “talks” in the couple relationship entail the ultimate site for this meaningful making of social life. Through conversation, unclear future images can be sharpened, at the same time as the past is reinterpreted in accordance with the couple’s present construction of reality. However, this construction is a fragile enterprise that requires constant maintenance. It is important to “talk through” and at “many times” (Berger, 1977, p. 38).

According to Berger and Kellner (1964), a joint child can strengthen the fragile construction of reality and become the subject of further dialog. At the same time, a child often brings new challenges. Feeling “time-squeezed” (Miller, 2017, p. 147) and not having enough space to talk often increase with the practices of parenthood (Fox, 2009). If the nomos cannot be maintained as before, this is probably an explanation for both changes in the couple’s attitudes and new strains between the parties. That Berger and Kellner do not address this theme to any greater degree may testify how theory always is situated in social context (Isaksson, 2020). Despite this, the theory is useful in understanding how couples negotiate collective narratives and social injunctions regarding the organization of family relationships. Moreover, the perspective may explain why ideas about parenting in a prenatal phase persist, or are changed, in the postnatal practices. In this article, the theory is used to explain the need for communication in order to establish belief and trust in an existential imperative, which arises with the realization that one’s everyday life and family relationships will soon be fundamentally changed.

## Materials and methods

This article is part of the research project *Ideals and practices of gender equality among parents in blue- and white-collar jobs. The role of the Swedish parental insurance*. Besides survey data, the project’s empirical material consists of longitudinal qualitative interviews with first-time biological parents in Sweden, collected in the prenatal and postnatal phases, with the aim of examining how ideas of justice and equality develop while becoming parents. The interviews during the prenatal phase, which is in focus here, took place between fall 2022 and spring 2023.

As this article focuses on the existential imperative of soon – but not yet – becoming a parent, and on the accounts of perceived uncertainties are mitigated, I only present data from the prenatal interview. For this first interview, the idea was that the pregnancy would not be in its infancy. The person with the longest distance to birth was in the fifth month of pregnancy while the interviewee who was closest to delivery gave birth the night after the interview, which meant that the father of the child had to be interviewed a couple of weeks later.

The interviewees were recruited mainly through advertisements in public spaces, newspapers, and social media, but also by trade union and workplace representatives. To date, 25 individuals have been interviewed, among them 11 couples. Three people were interviewed without their partner being part of the study, and although the other interviewees live together as couples, all interviews were conducted individually, based on the aim of analyzing the prospective mothers’ and fathers’ similar and different descriptions of the common process.

Among the interviewees, 24 defined themselves as heterosexual and one as transsexual. Three of the interviewees grew up in a European country other than Sweden. The interviewees’ median age was 33 years (mode: 30 years), with a range of 27 to 45 years with one outlier, an expectant father aged 58. Including this narrative in the sample as a “deviant case” (Platt, 2000) was a way of understanding the significance of age concerning different perspectives on family and gender relationships, and the role of expectations and communications [cf. Martin and Théry (2001)]. However, this expectant father’s perspective on these themes did not appear to differ from those of the younger prospective parents.

In Sweden, the gender equality policy has long aimed to develop equal divisions in paid work, household chores, and childcare, and the parental insurance has here been a key tool. Today, 90 of the 480 days of parental leave benefit are reserved for each parent. In 2022, women accounted for 70 percent of the take-up of Swedish parental benefits (Swedish Social Insurance Agency, 2023). However, highly educated parents with high income share parental leave benefit days more equally than do low-educated parents with low income (Duvander and Wiklund, 2020). Regarding the interviewees’ employment status, 20 have permanent jobs, two are students, one is temporary employed, and one is employed on an hourly basis, and one is on sick leave. Seven of the interviewees work in blue-collar jobs. Based on the research questions, the qualitative interview method aims to grasp a spectrum of experiences (Charmaz, 2014). In this respect, it constitutes a weakness that the qualitative data is dominated by the perspectives of white-collar workers. However, the data suggests that future fears and strategies for moderating them do not significantly differ according to different class capital; but as we will see, the metaphors that are used may differ.

The interviews were recorded and transcribed verbatim. To protect the interviewees’ integrity, the data has been de-identified; in the empirical section, I indicate only whether the speaker is an expectant mother or father. The interview data has been analyzed in mainly four stages: In an initial reading the context and meaning of the interviews were overviewed; then, in a second reading, this moved into close readings sentence by sentence. The third stage involved capturing the key metaphors and terms that represented the wider meaning of the sentences, which then became benchmarks for the fourth stage’s more abstract, theoretical level of interpretation (Charmaz, 2014). However, searching for key meaning structures is not the same as looking for a coherent logic in interviewees’ stories. Rather, qualitative analysis always involves paying attention to the complexity that the data offers through contradictions and ambiguities in the statements (Sennett, 2006).

The research methodology used in the article is phenomenological. Thus, the analytical focus is on how parenthood comes into being in relation to the environment [cf. Fox (2009)], with the past and future interacting in the contemporary prenatal situation. The analysis is primarily concerned not with “uncovering” hidden power relationships but rather with understanding the interpretive worlds of the expectant parents. I analyze how the interviewees try to make sense of parenting through accounts about their relationship conversations. Descriptions, metaphors, and details have guided the analysis in the attempt to comprehend their everyday life (Berger and Luckmann, 1966).

Moreover, the analytical focus has been on meaning and existential themes [cf. Douglas (2010)], and how existential concerns and thoughts about the past and the future become more present in the situation of expecting a first child [cf. Jackson (2005)]. This methodology, with the aim of analyzing how contemporary experiences are related to narratives of the past and future, is widely used in the holistic approach of life history method (Bertaux, 1981), in the methodology of timescapes (Holland, 2011; Neale et al., 2012), life course theory (Elder et al., 2003), and the social biography analysis [cf. Braidotti (1994), Douglas (2010), and Bengtsson and Flisbäck (2021)]. In these methodologies, the transition to parenthood is often examined as process over time, in relation to social structural and institutional conditions [see, e.g., Thomson (2010), Miller (2015), Lévesque et al. (2020), Grunow and Evertsson (2021), and Ranta et al. (2023)]. Although the analysis in this article is inspired by these methods, it is founded in data consisting of a cross-section in time, focusing on the participants’ thoughts just before becoming parents. This means that temporality is not used as a tool for analyzing how identities, relationships, and perspectives are changed or stabilized when the subject is moving from different social contexts at different times [see, e.g., Holland (2011) and Neale et al. (2012)]. What I analyze is the prenatal situation as an existential imperative giving rise to concerns and a new, complex experience of time. This is the first empirical theme to address.

## Results

### Anticipation of an uncertain future: the upcoming parenthood as an existential imperative

Characteristic of the life situations that Jackson (2005) aims to capture with the concept of existential imperative is that the situation of being “betwixt and between” is experienced as both open and uncertain. For the future parents this could be experienced as expectations of a changed everyday life, which will include challenging priorities. At the same time, the future parenthood is difficult to overview, and the images seem to be blurred:

**Expectant mother:** I think about what everyday life will be like… If this will change who I am as a person and my partner, if we’re going to prioritize things in a different way, or how this will impact us.

As Jackson (2005) underlines, openness often implies the possibility for a “better” future, while at the same time raising concerns and involving risk-taking. When the father-to-be below contemplates the future, he considers how a new stability could be arranged and what aspects of the past need to end:

**Expectant father:** It’s going to be tough to get everyday life together, with work and sleep, and it also feels very important to get into routines and have time to take care of yourself and exercise, and have time for each other, and have peace and quiet at times. Getting all those parts together.

Regarding the future parenthood, the interviewees express concerns that the practices of their new and old lives will be difficult to reconcile. These fears are accompanied by warnings from others about how parenthood implies giving up part of one’s previous life. Friends testify that it is difficult during the first period of parenthood to find time for anything other than family activities:

**Expectant father:** You know, some sarcastic comments like “Yeah, here you go, and we won’t see you again, but okay”. I mean, because for them, it’s the end of their life. You know, you end your current life and start another life.

Existential imperatives are changes that may cause life to take a new crucial direction, meaning that one often leaves something behind. This implies a situation of also experiencing parallel beginnings and endings. In this way, the experience of time is accentuated: In the present, the past and future are noticeably co-existent. The father-to-be above has male peers who testify that an important part of “life” ends with parenthood, but the question is what will take its place. I will look at this subject by first describing three future concerns identified by the interviewees.

### Three main concerns about new family relationships

Expecting a child can be interpreted as the beginning of what David Morgan (2011) calls *family practices*. It concerns both challenging and adapting to a new life in the frame of one of the most important institutions where social life is constructed: “the family” (Berger and Kellner, 1964; Berger, 1977). In this way, family practices can be said to begin already in the prenatal situation. This is illustrated above by the interviewees’ changed approach to time, with the future having become more present than before while the picture of what is to come is unclear. However, they express some concrete fears that correspond with research on main difficulties in the postpartum period: forming a new relationship with one’s child, one’s partner, and oneself (Lévesque et al., 2020).

*The first concern* that the interviewees express relates to the relationship they will have with their future child and taking on the task of caring for, and developing a good relationship with, the child. They explain that this uncertainty is based on their lack of previous experience of similar responsibilities. One expectant mother says she is particularly concerned about whether she will be a good parent, which in her view includes an ability to hold back immediate anger when one’s child acts unpredictably:

**Expectant mother:** I don’t know what it’s like to be a parent. I’ve had animals. /…/But not a human being who has to start talking and develop language and emotions. /…/Will I be a good parent, who doesn’t scream and get angry whenever the child does something they shouldn’t?

Thinking about the needs of the future child leads to concerns about what the future relationship will require of the prospective parent. The interviewees’ thoughts on how they will act, feel, and think can be understood as embracing a new identity as a mother or a father [cf. Miller (2005, 2017)]. *A second concern* arises from this, regarding a new relationship with oneself. This concern relates to the potentially limited scope for self-care and self-development when one becomes a parent. This may be underpinned by contemporary ideals, with paradoxical calls for parents to fully devote themselves to parenting while also maintaining their own interests (Lévesque et al., 2020). In relation to this, the interviewees reflect on friends’ experiences, with discouraging examples of new parents who can no longer engage in leisure activities. The expectant father below therefore believes it is essential to continue devoting himself to his own interests, even as a parent. However, he emphasizes that parenthood must involve preparation for the fact that life will take a new turn:

**Expectant father:** I have a lot of musician friends, and some disappear completely when they have children and can’t play music anymore and don’t have time for it. Music is the biggest identity I have. It has to work somehow! /…/ But that’s not the goal, anyway; the goal is to have children because you want to have children, not because your life should continue as usual.

The fear of not being able to pursue one’s own interests can concretely include not having enough time for an interesting job and thus “not being allowed to focus on oneself,” as an expectant mother puts it. “What will it be like to share my time?,” she asks. Another couple, both working in high-status occupations, express concerns that their careers will suffer when they become parents. However, the expectant father says they have tried to mitigate this worry by making a joint decision that this should not happen:

**Expectant father:** We’re both at workplaces where we work a lot, and we both agree that just because we’re starting a family it doesn’t mean we’re giving up our careers.

In addition to uncertainty about their new relationship with their child, and the possibility to make room for their own needs, the interviewees express a *third concern* involving the risk that parenthood will negatively affect the couple’s relationship. The interviewees say they have good reasons for this concern as they observe their social surroundings, but also point out that household chores are already difficult to manage in their present everyday life [cf. Wasshede (n.d.)]:

**Expectant father:** I mean, it’s a huge responsibility. And it’s not like having a dog; it’s a big responsibility. And we know nothing about it. We don’t know how to share responsibility for those kinds of things. I mean, sometimes we just talk about washing the dishes. I mean, now, washing the dishes has no importance compared to having a kid. I mean, it’s a life-changing thing. /…/ Responsibility and challenges might destroy our relationship. I see it with my friends; some of them get divorced.

A negative effect on the couple relationship is often evident to both women and men after the birth of a first child (Doss et al., 2009). When the expectant father above describes his fear that parenthood will negatively affect the relationship, his ultimate concern is that the situation will result in divorce. However, the interviewees often articulate that the couple relationship is a way of alleviating this concern: “The relationship,” says a mother-to-be, “how are we going to make it good?”; she then points out that the relationship has been rather difficult over the “years we have had,” but that this has led them to “talk about and find ways to deal” with difficulties. This statement illustrates a pattern in the data whereby the interviewees believe they can find a functioning future in which their child’s needs are met, while maintaining a good relationship with themselves and their partner. Thus, in the present situation, problem-solving from the past is drawn on as a strategy for solving future challenges.

### Conversation: a trustful foundation for future family practices

To alleviate the three concerns they described, the couples refer to their ability to communicate in a sensitive and respectful manner. The father-to-be below points out how the couple’s communication skills not only provide trust in the present but also lead the couple forward. The *alpha and the omega* metaphor illustrates that communication is a crucial phenomenon that encompasses everything the couple’s relationship has been, is, and will be:

**Expectant father:** The good thing in our relationship is that we both have… Communication is the key; it’s the alpha and the omega for us – it’s very important to communicate. It’s the only way to get things done; it’s talking, and we’re good at that!

Although the interviewees may emphasize more emotional or intellectual aspects of what constitutes a “good” discussion, it always seems to involve talking *openly*, without prejudging or judging the content. “We find it interesting to discuss high and low,” expresses a father-to-be. Furthermore, open communication means that the partners are sensitive to each other’s need to ventilate questions about the world around them, or to express difficulties in their shared life:

**Expectant mother:** Trust and things like that; I’d say that we know each other well now, where you can also be open about how you think and feel. /…/ I feel extremely secure in our relationship.

As depicted above, “being open” when communicating leads to getting to know each other. *Trust* is developed when you believe you know the other as both a person and a partner. The ability to talk openly is further described as *work* in the past that has counteracted both dissatisfaction and conflicts:

**Expectant mother:** We’re very good at talking to each other, about everything; but also, about things we’re dissatisfied with or if something’s difficult, so there are very few quarrels. /…/ This is something we’ve worked on over the years, and I think it makes us strong /…/ You don’t have to be afraid to say you’re unhappy or you think something’s difficult: we know we’ll be able to talk it through until we come up with a good solution.

Trust capital is an intellectual, emotional, and existential resource in which the ethic of reciprocity is central [cf. Mauss (2002), Théry (2003), Mauss (2002) Løgstrup (2020)]. In close relationships, trust capital can be generated when practices are shared and supportive conversations take a responsive form (Flisbäck, 2006, 2014). The concrete implication of this is that the parties need to have trust that the conversation will bring something positive but also, in times of difficulty, have trust that mutual “solutions” will come in due course. In the context of risks associated with situation of (soon, but not yet) becoming a parent, trust capital is therefore an invaluable resource. The possession of this capital counteracts existential uncertainty. “It’ll be a challenge, of course,” says an expectant mother, and goes on to explain that parenthood “will be something new, completely new, but /…/ I’m quite calm and secure in myself, and above all have trust in our relationship.” Thus, the trust created through the work of communication in the past constitutes both security in the present and confidence that the uncertain future of parenthood will be manageable.

### Conversation: assuaging uncertainty and making the future “real”

When the interviewees are faced with an open and uncertain future in which three relationships (with their child, their partner, and themselves) are to be (re)shaped, the couple’s generated trust capital seems to enable them to dare to believe in the future but also to enter the future “partnership” on reasonably equal terms:

**Expectant mother:** I hope and believe that we are thinking it’s companionship we are focused on during the initial period.

As stated above, regarding oneself as part of a team can be particularly important when facing the first period of parenthood, often depicted by friends (and in previous research) as the most stressful time. According to Berger and Kellner, in conversation couples shape a common understanding of the social world. This nomos may “assuage the ‘existential anxiety’” (Berger and Kellner, 1964, p. 16), not least when it is internalized in a homology consisting of shared perceptions of where the couple is going in the future and has been in the past. When talking about their relationships the interviewees sometimes used metaphors, for instance saying they shared a common “universe”:

**Expectant mother:** [We have a] shared vision of the framework, what life should look like, built on talking about exciting things. It feels like a shared brain and a shared universe.

As Berger and Kellner (1964) see it, a shared nomos requires a reconstruction of the past, a common narrative of what life was like before the two individuals met. Similarities in actions and thoughts can thus be portrayed as characteristics the individual always had but that have now found a harbor in meeting a soulmate. The interviewees describe themselves as very similar to their partner. Alongside good communication, this analogy is highlighted as a foundation of the relationships. “We think very similarly,” underlines an expectant father, “we understand each other, and I think this is a strength,” he continues.

In retrospect, the couples may have polished similarities to promote a contemporary homology between the two individuals. When the couples construct reality as comprehensible, similarity is central to building a common nomos (Berger, 1977) while dissimilarities can pose a fundamental threat. However, like most couples, the interviewees point out that they also differ from their partners to some extent, but they seem to (re)interpret these differences so that they are considered beneficial. When dissimilarity is portrayed in terms of *complementary qualities*, an idea is established that different interpretations lead the individual beyond their current understanding of the world:

**Expectant father:** Back each other up, complement each other, where the other is… not deficient, but you have a different way of looking at things and it’s very refreshing.

The couple’s communication is used as a trustful foundation in the challenge of becoming first-time parent, a stability that may both produce and reproduce social order. Reconstructed as complementary characteristics, differences seem to become not only understandable but also desirable (Butler, 1993). This could be seen as part of a communicative balancing act [cf. Théry (2010)] in which the couple’s shared perceptions of reality are both developed and maintained. In other words, claiming to be very similar to but at the same time different from one’s partner is an additional piece in the overall construction of a shared nomos.

Both similarity and complementarity create confidence in the future, even if the constructions of difference may also underlie a division of labor in the later parenting practices. However, simply talking oneself into a homologous understanding of reality is not enough; to develop a long-lasting foundation, the conversations must be recurrent (Berger, 1977). This may explain why the interviewees, in addition to the *quality* of the communication, also point to the importance of *quantity* in establishing “good” conversations:

**Expectant father:** We talk quite a lot; every day we have a lot of communication. Some people might say “you shouldn’t talk your marriage apart”. I think quite the opposite: Talking is good for the marriage!

According to Berger (1977), even if talking things through gives rise to an ethos and a way of living, “the social construction of reality” is often an unconscious act (Berger and Luckmann, 1966). However, as the interviewees describe the couple’s common stance, it appears to be more about a conscious approach to life, which is both an end and a means of the couple’s practice of communication. As illustrated below, this *existential motto,* as I call it, promotes shared values and interests, but also a mutual “pace” and a similar view of human nature:

**Expectant father:** We have very similar basic values, which means that there are usually no hard walls on issues. /…/ We both grew up in Christian homes and have brought that with us. /…/ And then we have a lot of common interests. We both have the music, and we have the same tempo most of the time. And we have no demands. Neither of us drinks, neither of us really likes to go out; we want to be at home on the couch watching movies, that’s when we feel our best. We follow each other very well.

Studying the prenatal situation means making visible an awareness of change whereby the past and future are linked in order to conquer a parental role that has never been experienced before. Analyzing how couples develop a nomos, and what I refer to as a common existential motto, provides an understanding of why conversations are given a central role in the prenatal interviews. Moreover, the concepts help in the comprehension of how parents-to-be search for a foundation while facing their new future. In our empirical data, through communication, the couples express how they become synchronized in everyday life, an interplay that means that worldviews, interests, and practices – despite differences – are shared.

**Expectant father:** We’re pretty synchronized in how we see the world, how we see the relationship, how we see family life. I mean, we, when we talk about how to raise a kid or something, we don’t have dramatic, extreme differences.

Existential motto is a complement to the concept of nomos and useful in capturing how social life is made intelligible through conversation in a more conscious way than Berger and Kellner (and Luckmann) allow for. The concept existential motto, sheds light on the last aspect of the social construction work; i.e., when the “real” becomes internalized in the individual and constitutes an orientation for the future. As described in the quotation above, the couple’s shared nomos includes ideas about future family practice such as childrearing, as well as joint dreams of the approaching family life [cf. Ranta et al. (2023)]. In other words, if nomos concerns the social reality that we jointly construct as true and “real,” existential motto concerns the part where the common reality is transferred into the direction for the future acting of the individual.

### Conversation: a democratic shield against future conflicts

Examining how communication in a couple relationship is used to construct a common nomos, which is further internalized as an orientation toward the future in terms of an existential motto, helps us gain comprehension of how couples mitigate concerns about their upcoming parenthood in the prenatal situation. However, with the use of these concepts we also may see how future family practices are in harmony or dissonance with policies and ideals of gender equality and parenthood. In this way, the concepts of nomos and existential motto provide an understanding of official family policies’ legitimacy among parents expecting a child, which I define in terms of an *existential legitimacy*. This concept involves how social ideas, norms, and values become understandable, “real,” and meaningful through conversations in couple relationships and, moreover, are internalized as an existential motto that gives rise to concrete ideas, such as how the couple’s future parental leave should be allocated:

**Expectant mother:** There was no major discussion. I said “I want the whole year”, and then my husband said “I want five months off”. So it was very simple. There was never any discussion, we just talked frankly.

**Expectant father:** We talked about it, and I said I want to be on paternity leave as much as I can. My wife thought that was positive, that I was so interested in being on parental leave as much as I want and can be. And because we have such straightforward communication with each other and talk to each other a lot, there were no problems when we came to that conclusion.

Similar to the expectant parents above, the other interviewees justify the allocation of future parental leave as “fair” as the plan is developed in communicative interaction based on openness and respect. According to the interviewees, in order to offer stability, faith, and trust, conversations must not be calculating, and the goal will never be to push one’s own perceptions and agendas through:

**Expectant father:** That there’s an open communication, before things become too much of a problem, and that it’s not filled with calculation or passive aggression, or a lack of trust that may eventually lead to a lack of security.

Gender equality (through sharing paid work, childcare, and household responsibilities) is described as an ideal in the Swedish society [see, e.g., Björnberg and Kollind (2005), Esping-Andersen (2016), and Björk (2017)]. However, when the prospective parents express thoughts on equality they primarily refer to the couple’s communication as the crucial tool for shaping democratic interactions and equality in everyday life. For instance, when the interviewee above depicts calculating conversation as the opposite of open and trustful communication, it is a reminiscent of Habermas’s (1987) vision of *deliberative democracy.* A distinction similar to Habermas’s is made between a *goal-oriented* approach and *responsive* communication. When interviewees also emphasize that the goal of communication is to achieve *consensus*, further similarities can be seen to Habermas’s idea of a communicative rationality, based on respect for the other’s arguments:

**Expectant father:** The fact that we, that we’re listening and… Which means we get along very well. There’s very rarely any major bickering.

As portrayed by the interviewees, they seem to see conversation as a shield against conflicts. Conversation stands in contrast to everyday “bickering”, meaning that consensus as a communicative goal also requires knowledge of when it is worth to stop talking. The words of the expectant mother below illustrate how communication, alongside open conversations, includes knowing when to leave aside disagreements that may not lead to consensus:

**Expectant mother:** I still think we’ve gotten better at solving it faster. Previously, there could be quite long, drawn-out arguments that in turn led to quarrels. For really small things. But now I think we’ve gotten a little better at saying “No, that’s enough! This is what I thought, this is what you thought. We don’t agree on this matter, so let’s move on!”

The quote illustrates how skills in communication seem to involve a flexibility in which the partners set aside their own agendas in favor of a listening that offers opportunities to move beyond their current personal views. This could be interpreted as a *renunciation* whereby the parties give up their own premises and interpretive prerogatives in order to gain something new (Carlsson and Flisbäck, 2024):

**Expectant mother:** If you think about how we organize our lives together, he’s not super principled and I wouldn’t say I am either. We’re very pragmatic and that makes life much easier.

Consequently, communicative consensus includes being open to conversing, but also an ability to understand when to “keep quiet.” This also may indicate that one’s confidence in one’s ability to work out difficulties with the other person is so strong that nothing more needs to be discussed. This was the case for the expectant mother below, when her husband promised her “time of her own” in the future so that even as a mother she could maintain her relationships with her friends:

**Expectant mother:** My husband told me this: “Whenever you want, you’ll be able to see your best friend and I’ll take care of the baby.” So, we very much agree on that too, without discussion. We have very little discussion in this family, we’re so interconnected.

Through conversation, this couple has developed ideas about how future family practices can take shape, including concrete strategies for counteracting future loss of autonomy. In this way, the communication is based on both conversation and an affirmative silence whereby “without discussion” indicates that both parties agree.

### Including the future child in the democratic conversation

While Berger (1977) points to the role of conversation in couple relationships (“marriage”) when constructing and maintaining the social reality, Habermas claims that responsive communication in close relationships is the fundamental source of citizens’ democratic approach. Consequently, Habermas argues that *how* we communicate with others is crucial for democratic development in modern society. Are conversations based on compromises or is the goal to *enforce power* and *unilateral influence*? While the latter is goal-oriented and the dominant form of communication in the *systems* – i.e., the logic of the state and the market – the former is based on the form of communication in the *lifeworld*, i.e., the close relationships in everyday life.

In the interviews, the democratic practice of conversation is expressed both as an end and a means to achieving sustainable family relationships. In addition, communication as a democratic ideal can become a guide for approaching one’s new, fuzzy, future relationship with one’s child. The metaphor of a dining table is sometimes used to depict the future child as part of the democratic family practice, based on equal space for speaking and listening. However, although the democratic ideal is the same, the representation may differ depending on the class capital. While the first of the expectant fathers below, a lawyer, has just described an intellectual academic conversation taking place at the dinner table, the other one, a caretaker, points to family gatherings at which “children’s tables” are the opposite of his and his wife’s ideal of having a communicative democracy in family practice:

**Expectant father:** Sitting at the dinner table, for me it’s that the family is a unit and that you always… Everybody knows we’re a team and that those of us in the family are there for each other.

**Expectant father:** Letting the child feel that they’re a part of the pack, the family /…/ It’s very important for a child to feel included, to be involved in decisions and in family events. Not putting the child at a separate table at parties.

The interviewees express a democratic ideal involving open, regular conversations based on recognition, freedom of expression, and consensus. If the family relationship is shaped in this “democratic” way, the prospective parents consider it equal and fair. Therefore, it is also essential to include the future child in the couple’s communicative practice. Here, it is necessary to combine the view of Berger, concerning the role of conversations in everyday life, with the perspective of Habermas. Namely, allowing the child to be part of the democratic conversation in family relations is a way of both classifying blurred, uncertain future images of what one’s relationship with the child might involve and developing democratic ideas and practices. Thus, in the existential imperative of expecting a first child, ambiguous images of future parenting become more concrete, comprehensible, and ordered [cf. Berger and Luckmann (1966, p. 98)], at the same time as one’s belief in “democratic family talk” is developed and strengthened.

## Discussion

### Conclusion

As an existential imperative (Jackson, 2005), expecting a first child entails experiencing the beginning of a new life while ending another, with emerging concerns about what losses the new future will bring. From a phenomenological perspective, we have seen that these concerns relate to the transformation of one’s family relationships, i.e., the changed relationship with one’s partner, oneself, and one’s future child [cf. Lévesque, et al. (2020)]. To alleviate these concerns, the interviewees relate to past experiences of solving difficulties through constructive and regular conversations.

The *first aim* of this article was to understand the main concerns of the interviewees in order to explain why the conversation pattern (established in the *past*) plays such a decisive role in mitigating the *present,* prenatal uncertainty about *future* parenting. In relation to this, some *basic elements* have been identified as essential to believe when it comes to the role of the conversations in addressing concerns. These respond to the *second aim* of the article: to explore how reality is concretely made understandable in the conversational practice. It is a matter of *frequent*, *open*, *consensual conversations* in which self-interest sometimes needs *renunciation* in order to *avoid conflict* and the characters’ *similarities* are emphasized at the same time as their *differences* are seen as *complementary qualities*. All in all, these concrete elements seem to be crucial in order for conversations in long-term couple relationships to provide the foundation on which the “fabrics” of social reality are constructed [cf. Berger and Kellner, 1964 and Berger and Luckmann (1966)].

In order to meet the first aim of the article, an answer is needed regarding what specific and concrete role the conversation plays in the existential imperative of expecting one’s first child. It appears that *the couple’s conversation seems to mainly fulfill three functions*, all of which support a belief that future parenthood will be manageable. *Firstly*, close, respectful conversation creates *trust capital*. This happens through an empowering experience of not being left alone in the human will to communicate and experiencing that one’s trust is not exploited or treated with indifference [cf. Løgstrup (2020)]. When conversations also bring about concrete support in everyday life, trust capital is generated, allowing individuals to dare to venture into the open and uncertain world of parenthood.

*Secondly*, the couple’s communication can be seen as an important practice that *externalizes* and *objectifies* ideas about – and experiences of – family practices as “real.” Through communication a shared reality is formed, which counteracts the ambivalence and existential uncertainty of the situation [cf. Berger (1977)]. Accordingly, reality is conquered, negotiated, and comprehended, and a common *nomos* (Berger and Kellner, 1964) is formed. When this is *internalized* in the individual as an *existential motto*, the couples are led toward the future of parenthood (cf. the second aim of this article: to conceptually develop Berger’s perspective).

*Thirdly*, the communication in the couple relationships seems to justify the formation of the future family relationships as democratic when the interviewees refer to their plans as having arisen in consensus through open and responsive conversation. Moreover, the interviewees hope that the relationship’s democratic communication will also include the child, and thus mitigate difficulties in their new family practices. Here, it must be stressed that I neither attempt to determine whether the interviewees’ conversational practices *are* democratic nor examine whether the conversations lead to an equal distribution of paid labor, childcare, or household chores [cf. Wasshede (n.d.)]. Rather, I have shown *how* communication is presented in a way that is similar to the idea of deliberative democracy (Habermas, 1987). *When dialogue is regarded as democratic, one’s belief is enhanced that future family relationships will be built on a trustful, stable, and fair foundation*. However, even though the couple’s communication is considered a trustworthy democratic tool, this stability offered at the micro level may simultaneously prevent changes at the macro level. This complexity is important to consider when developing a gender equality policy that is to resonate with people’s everyday lives; this argument is developed below in relation to future research.

### Future research: trustful conversations and existential legitimacy for welfare policy

The importance of communication in close relationships in building trust and mitigating uncertainty in everyday life is a recurrent sociological theme. As an influential institution, conversations in “marriage” – or a long-term couple relationship – are said to have increased in modern society, while the authority of other institutions has decreased [see, e.g., Berger and Kellner (1964), Finch and Manson (1993), Giddens (1992), and Martin and Théry (2001)]. This is accompanied by a development in which (heterosexual) relationships have been given a different meaning as legal rights and the expansion of the welfare state have sought individual independence and defamiliarization (Beck and Beck-Gernsheim, 2002; Goldscheider et al., 2015; Esping-Andersen, 2016). When practical, financial, and emotional support are no longer given obligations but are rather negotiated in the conversations of everyday life, there is a need for studies that not only highlight *how* and *why* this is done but also point out *the democratic significance of these everyday conversations* [cf. Deutsch (2007) and Giddens (1992)].

Conversations in couple relationships mitigate concerns and build trust for future parenthood, but also balance expectations in an interaction that embodies practical ideas about democracy and equality. A key point in this article is that this latter aspect becomes particularly important in existential imperatives when issues of life, time, and meaning are brought to a head. In this way, the existential dilemmas of the individual, and the couple’s communicative interaction in dealing with them by developing a common nomos and an existential motto, can be a way to understand how the micro level of social life is connected to the macro level [cf. Flisbäck and Bengtsson (2024)]. Or to put it differently: Examining how concerns (arising in existential imperatives) are handled through conversations in long-term couple relationships tells us something crucial about how *citizens develop an existential legitimacy in the face of welfare state policy*. As Habermas (1996) once argued, laws and policies of the welfare state can never be democratic if citizens experience them as imposed. In order to become a social resource and be experienced as “real,” family and gender equality policy (such as parental leave insurance) must have a resonance in the communication of the lifeworld and the ability to impact the public sphere.

The concepts developed in this article, existential motto and existential legitimacy, may highlight the main place where welfare state policy is made “real”; or conversely, the place where the policy is dismissed as “completely detached from reality.” However, in order to be applicable in this way, the results and conclusions need to be analyzed from a critical standpoint as well. When forthcoming family practices are made an objective reality via the couple’s conversation, this involves both the *production* and *reproduction* of norms and *social power relationships* [cf. Berger (1977), Fox (2009), and Miller (2017)]. With the aim of consensus, and with the means of “renunciation,” there is a risk that communication in long-term relationships will consolidate the interests of the stronger party [cf. Fraser (1990)]. In this way, the conversational practice may reproduce social inequality among women and men and counteract the welfare policy goal of equalizing power relations. According to Chantal Mouffe (2013), under the banner of consensus, deliberative democracy silences the voices of the oppositional, as consensus is always based on the exclusion of difference.

Furthermore, like other forms of assets, trust capital can be exploited (Flisbäck, 2006). The generation of capital always implies the danger that the self-disclosure of trust will be taken advantage of [cf. Løgstrup, (2020)]. One party (often a woman) may instill support and confidence in the other (often a man) more often than receiving it in return [cf. Jónasdóttir (1991)]. However, highlighting the couple’s “deliberative democratic” conversation as the key to good family relationships not only involves the danger of concealing unequal gender relationships; there is also a risk that the widespread view that enabling parental practices are based foremost on the closeness of *two* people *staying together* will be reproduced.

When analyzing conversations in couple relationships as a basis for mitigating concerns in the transformation of family relationships, it is interesting that the interviewees underline trust but talk less about the love between them. Perhaps they have reached an insight similar to that noted by Illouz (2021), that love in the context of the late capitalist society always ends – at least if love is defined as Illouz defines it, as a strong sense of passion that is mainly used to strengthen the self. Although support, care, and benevolence are mentioned in the interviews (and are essential aspects of possessing trust capital), communication is highlighted as a more concrete, stable foundation that is necessary for the belief of parenthood. This result points to the need for further analysis of the impact of everyday communication in long-term relationships as an important social institution for constructing the social reality and building trust in the uncertain situation of becoming a parent. Moreover, the article’s findings point to a need for studies that analyze both the couple conversation, by taking it into account as the central site for developing an existential legitimacy for welfare state policy, as well as how democratic approaches are particularly developed in existential imperatives, such as expecting a first child.

## Data availability statement

The original contributions presented in the study are included in the article/supplementary material, further inquiries can be directed to the corresponding author.

## Ethics statement

The studies involving humans were approved by the Swedish Ethical Review Authority, no. 2022–00471-0. The studies were conducted in accordance with the local legislation and institutional requirements. The participants provided their written informed consent to participate in this study.

## Author contributions

MF: Writing – original draft, Writing – review & editing.
